# Phylogenetic analysis of the core histone doublet and DNA topo II genes of Marseilleviridae: evidence of proto-eukaryotic provenance

**DOI:** 10.1186/s13072-017-0162-0

**Published:** 2017-11-28

**Authors:** Albert J. Erives

**Affiliations:** Department of Biology, University of Iowa, Iowa City, IA 52242-1324 USA

**Keywords:** Chromatin, Origin of eukaryotic chromatin, Core histones, Nucleosomes, DNA topoisomerase II, Eukaryotic replisome, Marseilleviridae, NCLDV, Archaea, CRISPR

## Abstract

**Background:**

While the genomes of eukaryotes and Archaea both encode the histone-fold domain, only eukaryotes encode the core histone paralogs H2A, H2B, H3, and H4. With DNA, these core histones assemble into the nucleosomal octamer underlying eukaryotic chromatin. Importantly, core histones for H2A and H3 are maintained as neofunctionalized paralogs adapted for general bulk chromatin (canonical H2 and H3) or specialized chromatin (H2A.Z enriched at gene promoters and cenH3s enriched at centromeres). In this context, the identification of core histone-like “doublets” in the cytoplasmic replication factories of the Marseilleviridae (MV) is a novel finding with possible relevance to understanding the origin of eukaryotic chromatin. Here, we analyze and compare the core histone doublet genes from all known MV genomes as well as other MV genes relevant to the origin of the eukaryotic replisome.

**Results:**

Using different phylogenetic approaches, we show that MV histone domains encode obligate H2B-H2A and H4-H3 dimers of possible proto-eukaryotic origin. MV core histone moieties form sister clades to each of the four eukaryotic clades of canonical and variant core histones. This suggests that MV core histone moieties diverged prior to eukaryotic neofunctionalizations associated with paired linear chromosomes and variant histone octamer assembly. We also show that MV genomes encode a proto-eukaryotic DNA topoisomerase II enzyme that forms a sister clade to eukaryotes. This is a relevant finding given that DNA topo II influences histone deposition and chromatin compaction and is the second most abundant nuclear protein after histones.

**Conclusions:**

The combined domain architecture and phylogenomic analyses presented here suggest that a primitive origin for MV histone genes is a more parsimonious explanation than horizontal gene transfers + gene fusions + sufficient divergence to eliminate relatedness to eukaryotic neofunctionalizations within the H2A and H3 clades without loss of relatedness to each of the four core histone clades. We thus suggest MV histone doublet genes and their DNA topo II gene possibly were acquired from an organism with a chromatinized replisome that diverged prior to the origin of eukaryotic core histone variants for H2/H2A.Z and H3/cenH3. These results also imply that core histones were utilized ancestrally in viral DNA compaction and/or protection from host endonucleases.

**Electronic supplementary material:**

The online version of this article (10.1186/s13072-017-0162-0) contains supplementary material, which is available to authorized users.

## Background

The Marseilleviridae (MV) are a distinct family of viruses within the nucleo-cytoplasmic large DNA viruses (NCLDV) [1–3] with eukaryote-like core histone genes [4, 5]. These histone genes are unusual in at least three ways: (i) most of the histone domains are orthologous to eukaryotic core histones (H2A, H2B, H3), but one (h) has been weakly assigned to the single archaeal histone clade [5]; (ii) the core MV histone domains are “fused” into divergently transcribed doublet genes, thus encoding forced h-H3 and H2B-H2A heterodimers; and (iii) MV histone proteins were reported found in the virus particles of Marseillevirus, thus suggesting MV nucleosomes function in the compaction, protection, and/or regulation of their large viral genomes [4].

The MV core histone repertoire is unlike archaeal genomes [6–8], which typically encode a generic histone that forms obligate homodimers and a tetrameric nucleosome that protects ~ 60-bp DNA [6]. However, more recent structural studies indicate the ability of archaeal histone homodimers to form a variety of different multimeric complexes and nucleosomes with eukaryote-like DNA compaction [9]. Nonetheless, archaeal histones are typically short peptide sequences 65–69 amino acids in length [7] and lack the N-terminal histone tails of eukaryotic histones, which are epigenetically modified by covalent attachments of acetyl and methyl groups at conserved lysine residues [10]. Thus, the presence of genes for forced dimers of eukaryote-like core histones in MV genomes is remarkable and raises the question as to whether they are more closely related to core histones of particular eukaryotic lineages or to particular core histone variants. For example, is the H2A moiety of the MV H2B-H2A fusion protein more closely related to canonical H2A or to H2A.Z, each of which is highly conserved across eukaryotes?

Genes for linker histones (H1/H5) have not yet been found in any of the six known Marseilleviridae genomes. These include the 368-kb Marseillevirus genome [4], the 374-kb Cannes 8 virus genome [11], the 346-kb Lausannevirus genome [5], the 386-kb Insectomime virus genome [12], and the 380-kb Tunis virus genome [13]. About 300–400 genes are shared among the Marseilleviridae viruses, with about ~ 600 protein-coding genes present in the pan-MV genome [14].

Initial phylogenetic analysis of the Marseillevirus and Lausannevirus histones genes revealed a “challenging” perspective of MV histone origins, evolution, and relationships to eukaryotic histones [5]. Investigation of the origin and makeup of the MV histones might be relevant and/or informative to understanding the origin of eukaryotes, which exceed prokaryotes in cellular complexity, compartmentalization, and their sophisticated chromatinized genomes [15, 16]. MV chromatin might constitute an independent model for the evolutionary chromatinization of a genome if the MV histone genes were acquired from an unknown eukaryotic group and were maintained for eukaryote-like chromatinization. Alternatively, the MV core histones might even be derived from a stem-eukaryotic lineage. If so, these stem-eukaryotic remnant genes within MV genomes could provide information on the evolutionary origin of eukaryotes.

To investigate the evolutionary origins of MV histones, we consider recently sequenced MV genomes (e.g., Insectomime virus/Tunis virus, Cannes 8 virus, and Melbourne virus) discovered since the Marseillevirus and Lausannevirus reports [11–14, 17]. We also considered both canonical core histones, which are unique to eukaryotes, and eukaryotic variants such as H2A.Z and CenH3/CENP-A. The H2A.Z histone is associated with promoter nucleosomes, which is likely the ancestral “genic” or gene-regulatory function conserved across eukaryotes and Archaea [8]. The cenH3 histones are fast-evolving H3-like eukaryotic histones associated with centromeric nucleosomes [18]. Centromeres are the chromosomal regions targeted by kinetochores, which are a eukaryote-specific innovation associated with their linear chromosomes and which function in proper chromosomal segregation [18]. While centromeric H3 variants are fast evolving and can be lost [19] and presumably replaced with co-opted H3 paralogs [20–22], inclusion of these variants could still be informative. Last, we also considered eukaryotic groups among the Discoba super-group, which phylogenetic analysis puts as the sister group of all other eukaryotes [23]. Within this group are the Excavates and in particular the kinetoplastid protists (e.g., Trypanosome parasites), which lack homologous components of the kinetochore, which assembles on centromeres [24]. Several excavates have divergent H3 variants utilized in non-centromeric regions such as telomeres [25], polycistronic loci [26], or in other non-canonical chromosomal structures [27, 28].

Here, we show that the MV genes encoding core histone moieties, and at least one other key component of the eukaryotic replisome, likely were acquired prior to the evolutionary diversification of eukaryotic core histone variants. First, we use phylogenetic analysis to place these core MV histone domains as sister clades to each of the four eukaryotic core histones. The fused H2B-H2A and H4-H3 genic configurations are understandable if the MV H2A and H3 clades are really out-groups to eukaryotic core variants (i.e., paralogy groups), which evolved specialized functions in the context of the eukaryotic linear chromosome and differently chromatinized regions (e.g., intergenic DNA, genic/regulatory DNA, and centromeric DNA). Genes encoding forced core histone heterodimers would be possible in the absence of core histone variants and the need for swappable, combinatorial interactions. Second, we show that other MV genes encoding DNA replisome components adapted to a chromatinized template, such as DNA topoisomerase II, also form a clade that is sister to all eukaryotic DNA topo II sequences. Third, the maintenance of these core histone fusions and DNA topo II genes in divergent MV genomes suggests that these genes are essential to the MV life cycle. Altogether, these results suggest that the study of the Marseilleviridae replisome may be informative for understanding intermediate steps in the origin of eukaryotes.

## Results

### MV core histone genes are proto-eukaryotic-like

**Fig. 1:**
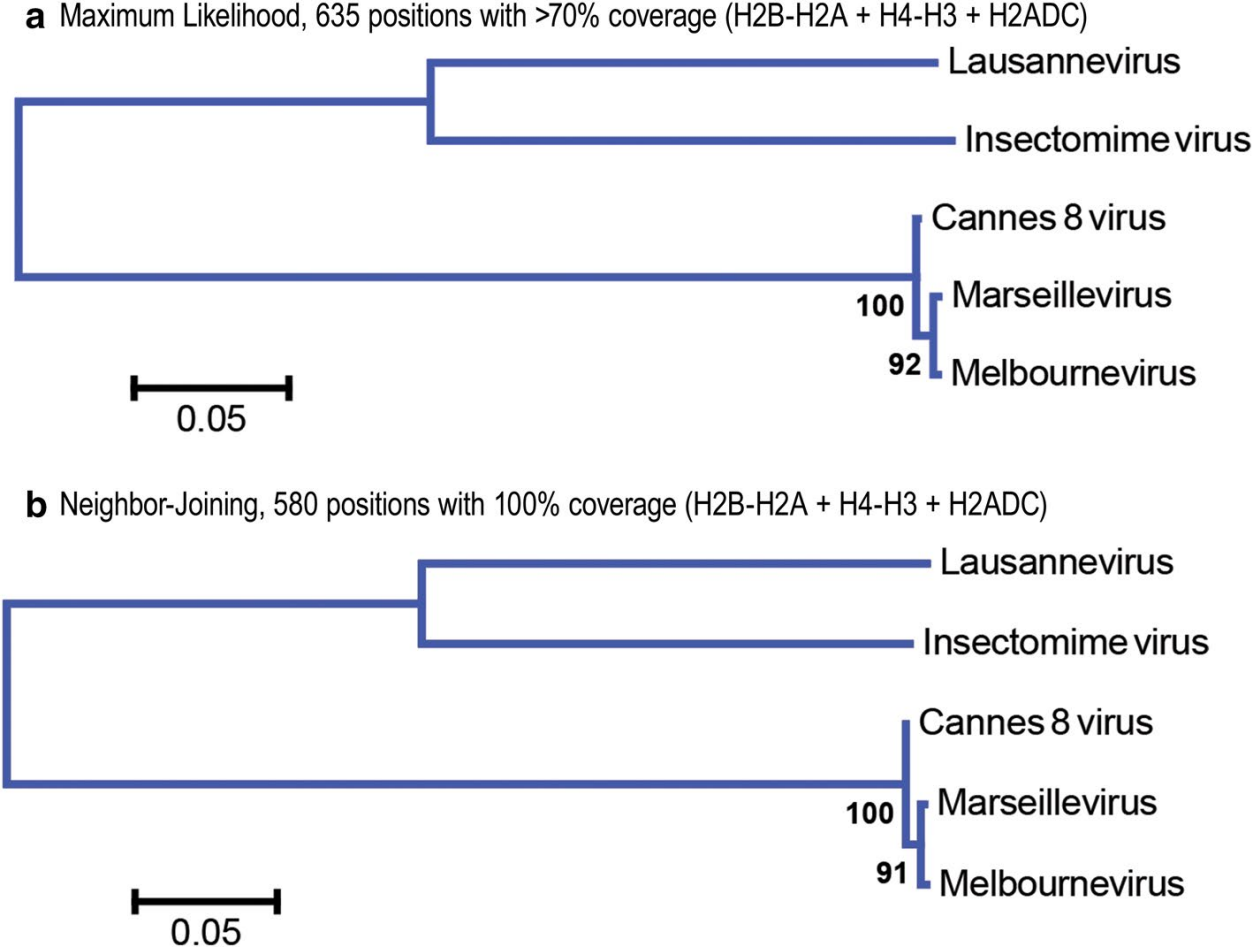
Evolution of core histone genes from Marseilleviridae (MV). Phylogenetic analysis of all known histone-containing proteins predicted to be encoded in Marseilleviridae genomes, including Lausannevirus, Insectomime virus, Cannes 8 virus, Marseillevirus, and Melbourne virus, shows that these are slow evolving. This analysis also shows that Marseillevirus, Lausannevirus, and the Insectomime viral repertoires are representative of the full divergence within the MV family and are used exclusively in the remaining figures. **a** Shown is a phylogenetic analysis of the concatenated histone repertoires of each available MV genome conducted by the maximum likelihood (ML) method with the Le and Gascuel [8] amino acid substitution model [55]. The optimal tree with the highest log likelihood is shown along with the percentage of trees in which the associated taxa clustered together in the bootstrap test of 500 replicates. A discrete Gamma distribution was used to model evolutionary rate differences among sites (five categories). Branch lengths are measured in the number of substitutions per site. All positions with less than 70% site coverage were eliminated leaving a total of 635 positions in the final dataset. **b** A phylogenetic analysis of the concatenated histone repertoires of each available MV genome using the neighbor-joining method [56] gives the same result as in **a**. The percentages from 500 bootstrap replicate trees in which the associated taxa clustered together are shown next to the branches. Evolutionary distances were computed using the JTT matrix-based method [57] and are in the units of the number of amino acid substitutions per site. The rate variation among sites was modeled with a gamma distribution (shape parameter = 1). All positions containing gaps or missing data were eliminated leaving a total of 580 positions in the final dataset. Both the ML and NJ trees were computed using a ClustalW-based alignment of the concatenated peptide sequences from the H2B-H2A, H4-H3, and H2A-domain-containing (H2ADC) genes found in all MV genomes, and the MEGA6 software package [53]

The Marseilleviridae genomes of Marseillevirus and Lausannevirus encode three histone-fold-containing proteins, two of which occur as a pair of divergently transcribed genes encoding histone doublets H2B-H2A and h-H3, where h is an ambiguous histone domain that groups either with the single archaeal histone clade (h), or else with the eukaryotic H4 core histone clade [5]. These three histone-encoding genes (H2B-H2A, h-H3, and H2ADC) are found in each of five available MV genomes (analyzed in Fig. 1) and collectively encode five histone-fold domains with at least one histone-fold domain (referred to as a core histone “moiety” in the fusion proteins) from each gene belonging to the “H2A” domain superfamily characteristic of eukaryotes and Archaea. Phylogenetic analysis of a concatenated alignment between all three H2A-domain-containing genes shows that using the subset of Marseillevirus, Lausannevirus, and Insectomime would accurately represent the full diversity of MV genomes (Fig. 1).

**Fig. 2:**
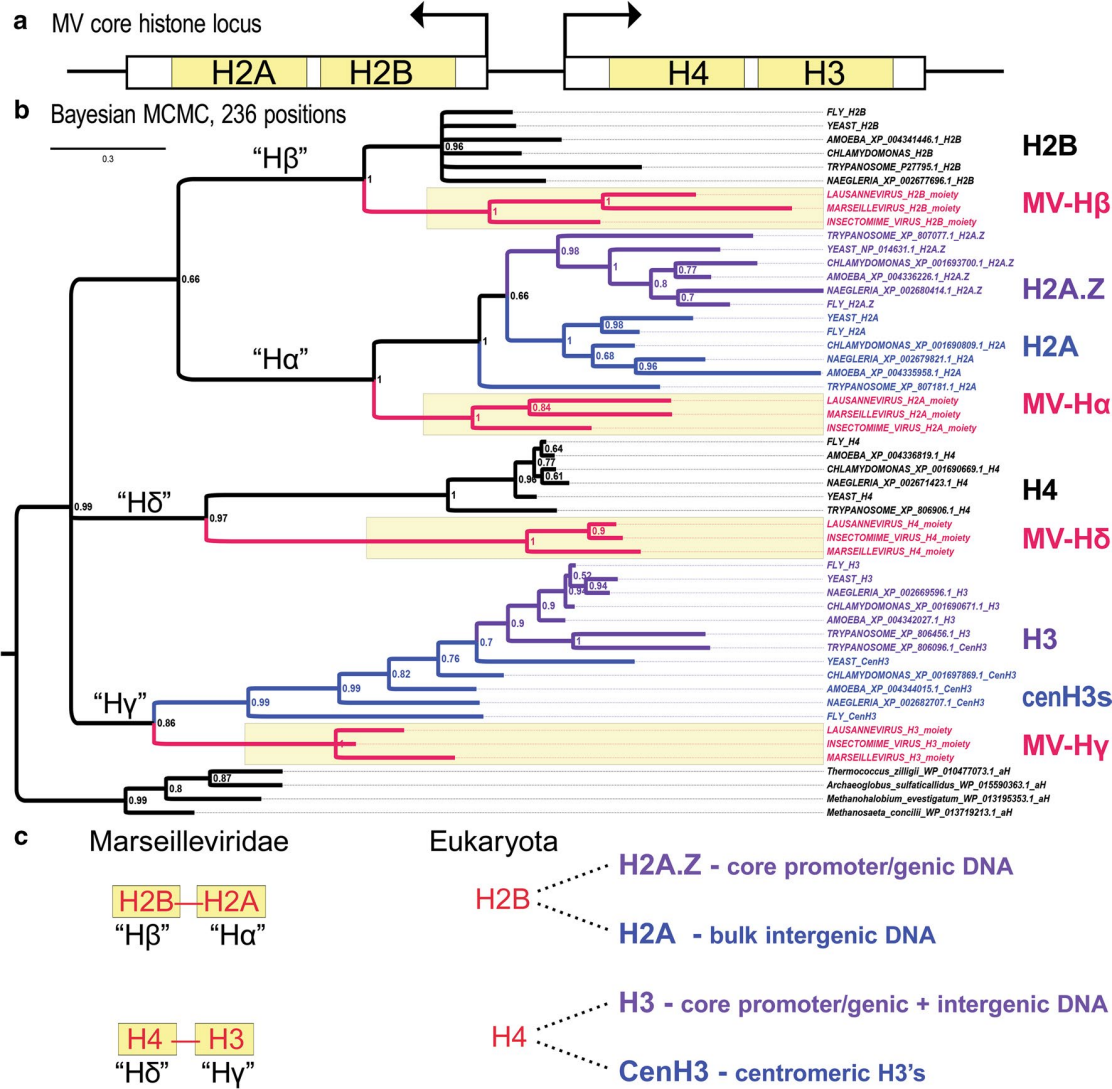
The MV core histone locus defines a full repertoire of basal eukaryotic core histones. **a** All MV genomes possess a pair of divergently transcribed histone doublet genes encoding a predicted protein with fused H2B and H2A moieties (H2B-H2A) and another predicted protein with fused H4 and H3 moieties (H4-H3). This gene pair thus encodes obligate H2B-H2A and H4-H3 dimers. **b** Bayesian MCMC-based inference shows that each MV core histone domain defines a well-supported sister clade (yellow highlighted clades with red lineages) to the eukaryotic core histone groups, including eukaryotic core variants for H2A.Z/H2A and H3/CenH3 (purple and blue clades within each core family). Posterior probabilities following 2,000,000 generations of sampling mixed amino acid substitution models with 25% burn-in generations and without Metropolis coupling (heated chains) are indicated at all nodes. The average standard deviation of split frequencies from two parallel runs was less than 1% (0.0076). This analysis indicated posterior probabilities of 80.5 and 19.5% for the Wag [58] and Blosum [59] amino acid models, respectively. All core histone clades from eukaryotes and MV viruses are grouped in a single super-clade of the core histones with a posterior probability of 0.99. Representative archaeal histones are grouped together in an out-group clade at the bottom. This analysis was conducted on an alignment using MUltiple Sequence Comparison by Log-Expectation (MUSCLE) [32]. **c** The above phylogenetic analysis suggests that that the basal core histones predate eukaryote-specific duplications and neofunctionalizations in the Hα and Hγ clades. Interestingly, some of these eukaryote-specific specializations are associated with intergenic nucleosomes (eukaryotic canonical H2As) or centromeric nucleosomes (cenH3s). Thus, the core basal histones, defined as Hα, Hβ, Hγ, and Hδ likely predate the evolutionary innovation of large, linearized chromosomes with centromeric pairing mechanisms, a late stem-eukaryotic innovation

To evaluate the intriguing MV core histone repertoire in light of more recent MV genomes [13, 14, 17] and in greater detail, we phylogenetically analyzed the four histone domains (“moieties”) of the obligate doublet genes (see Additional file 1 for sequences). The H2B and h moieties of the divergently transcribed histone doublet genes each occur in the N-terminal halves of the predicted proteins (Fig. 2a). Interestingly, this leaves the H2A and H3 moieties in their respective C-terminal halves and these correspond to the core histones associated with distinct functional variants, such as H2A.Z and cenH3/CENP-A.

We conducted Bayesian phylogenetic analysis [29–31] based on global alignments constructed via MUSCLE, MUltiple Sequence Comparison by Log-Expectation [32], and/or CLUSTALW [33] of the following: (i) the four separated MV histone moieties (Fig. 2a) from the three most diverged MV genomes of Insectomime, Lausannevirus, and Marseillevirus, as indicated by our phylogenetic analysis of all known MV genomes (Fig. 1). We also included eukaryotic sequences for key core histone variants including H2A.Z in addition to “canonical” H2A, and cenH3 in addition to canonical H3, as well as representative histones from Euryarchaeota.

We find that all four MV core histone domains form highly supported sister groups to the four core histone clades of eukaryotes (Fig. 2b). These results further resolve that the “h” MV histone moiety is allied to an H4 core histone super-clade and not the archaeal histone clade (0.97 support). Resolution of this cryptic histone domain as a *bona fide* H4 ortholog is consistent with its joined condition alongside its H3 partner moiety. Thus, the standard MV genomic configuration of a full core histone repertoire of forced H2B-H2A and H4-H3 doublets is remarkably similar to an anticipated basal/intermediate eukaryotic condition of fused histone doublets, which would have arisen by tandem duplications [34].

There is perfect support for an ancestral eukaryotic duplication that produced canonical (“bulk”) H2A and variant H2A.Z (compare purple H2A.Z and blue H2A sister clades in Fig. 2b) after MV core histone divergence. As H2A.Z retains the ancestral function of participating in the +1 nucleosome of genes at their core promoters as well the downstream genic nucleosomes to a lesser extent [8], we suggest that H2A may represent a neofunctionalized H2A.Z paralog that became specialized for bulk intergenic DNA. Thus, our results highlight both the possibility and utility of conceptualizing the stem-eukaryotic branch into pre- and post-variant core histone functions.

### Names for core histones related to intermediate proto-eukaryotic stages

Use of the terms “Hα,” “Hβ,” “Hγ,” and “Hδ” for the ancestral core histone variants that gave rise to both canonical and variant non-canonical eukaryotic core histones would help avoid the ambiguity and inappropriateness of naming the MV core histone genes after eukaryote-specific core histone variants. For example, we found an H2A-like moiety in one of the canonical MV doublet genes, but this moiety was found not to correspond exclusively to either canonical H2A or variant H2A.Z, but rather to a sister clade of the eukaryote-specific duplications, canonical H2A and variant H2A.Z. For this reason as well as for phylogenetic principles for histone naming [35], we refer to the H2B-H2A and H4-H3 doublet genes as the Hβ-Hα and Hδ-Hγ genes, respectively (Fig. 2c). Within this framework, Hα is the ancestral core histone ortholog that duplicated in the late stem-eukaryotic lineage to give rise to distinct H2A.Z and H2A paralogs, while Hγ is the ancestral core histone that flourished into canonical H3 and centromeric cenH3s-/CENP-A-like core histones (Fig. 2b).

As previously found by others [19, 34], we find no support for a single clade of eukaryotic centromeric H3 variants that excludes canonical H3 (Fig. 2b, latest ancestral node of all cenH3s is ancestor to canonical H3s as well). Nonetheless, our phylogenetic analysis supports a eukaryotic super-clade (0.99 support) that includes both the fast-evolving centromeric cenH3s and the canonical H3 clade to the exclusion of a sister clade of MV Hγ (Fig. 2b). This is consistent with repeated evolutionary co-option of eukaryotic H3s into centromeric roles occurring after divergence of MV Hγ.

In retrospect of this analysis, new eukaryotic requirements for combinatorial interactions between Hβ and H2A or H2A.Z, and between Hδ and H3 or cenH3s, likely demanded the evolution of singlet (non-fusion) H2B and H4 genes as well (Fig. 2c). Thus, these phylogenetic results suggest that the MV core histones likely predate the eukaryotic innovation of linear paired chromosomes with centromeres and substantial intergenic DNA.

### MV DNA topoisomerase II is eukaryote-like but is not assignable to any eukaryotic lineage

Components of the eukaryotic replication fork also interact with conserved eukaryotic machinery functioning in histone deposition and chromatin compaction. To investigate whether additional genes are conserved across the Marseilleviridae that might function in a replisome complex adapted to working with core histone-based nucleosomes, we considered all 127 annotated Lausannevirus proteins with known domains to see which are most conserved with eukaryotes. We conducted a BLASTP query of the *Saccharomyces cerevisiae* proteome and identified the DNA topoisomerase II (DNA topo II) homolog as the best match (1e–174 *E*-value, 1009 amino acid long protein encoded by yeast *TOP2*). DNA topo II is the second most abundant eukaryotic nuclear protein after histones and influences chromatin compaction and histone deposition [36, 37]. The major domain of eukaryotic DNA topo II is homologous to the archaeal DNA gyrase subunit B, which can provide an out-group sequence.

**Fig. 3:**
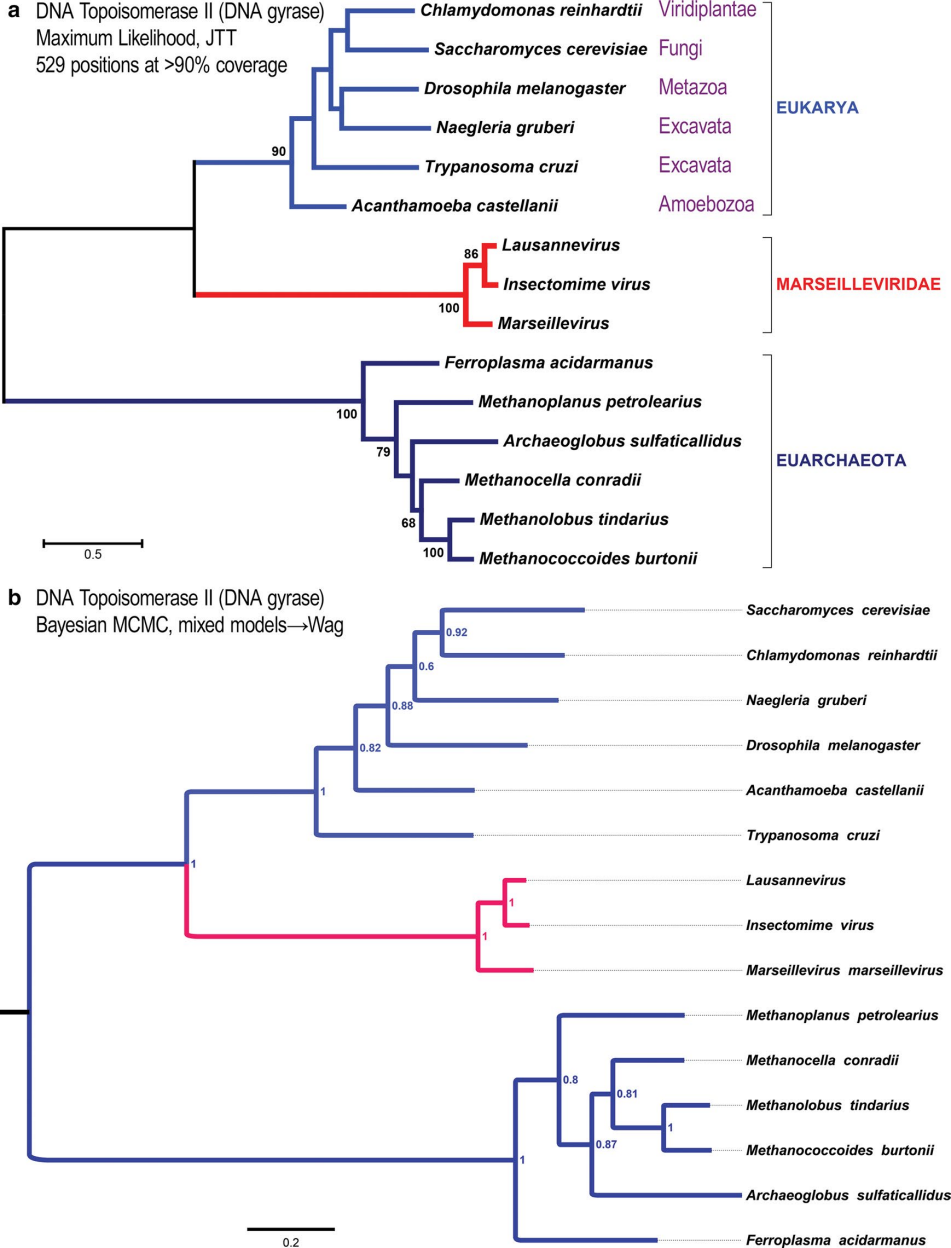
All MV genomes encode a basal eukaryotic DNA topoisomerase II protein. The presence of eukaryote-like nucleosomal chromosomes in MV is likely to require adaptations in the complexes operating at viral DNA replication forks. Analysis of MV genomes shows that the most conserved gene across MV and Eukarya is the gene encoding the large DNA topoisomerase II enzyme, which functions at the replisome. Together, phylogenetic analyses of the MV core histones and the MV DNA topoisomerase II show that these MV genes do not group within eukaryotes or Archaea. **a** Phylogenetic analysis by maximum likelihood on an alignment including all 529 columns with > 90% data from all taxa and the JTT substitution matrix [57]. Support values are from 500 bootstrap replicate data sets from 529 alignment columns that remained after a threshold cutoff of 90% data was applied. Only percent bootstrap replicate values > 60% are shown. **b** Phylogenetic analysis by Bayesian MCMC using the same alignment as in (**a**). 2,000,000 generations of parallel runs with final average standard deviation of split frequencies = 0.0129 with 25% burn-in generations after sampling mixed amino acid models. Final generations sampled the Wag substitution model

**Fig. 4:**
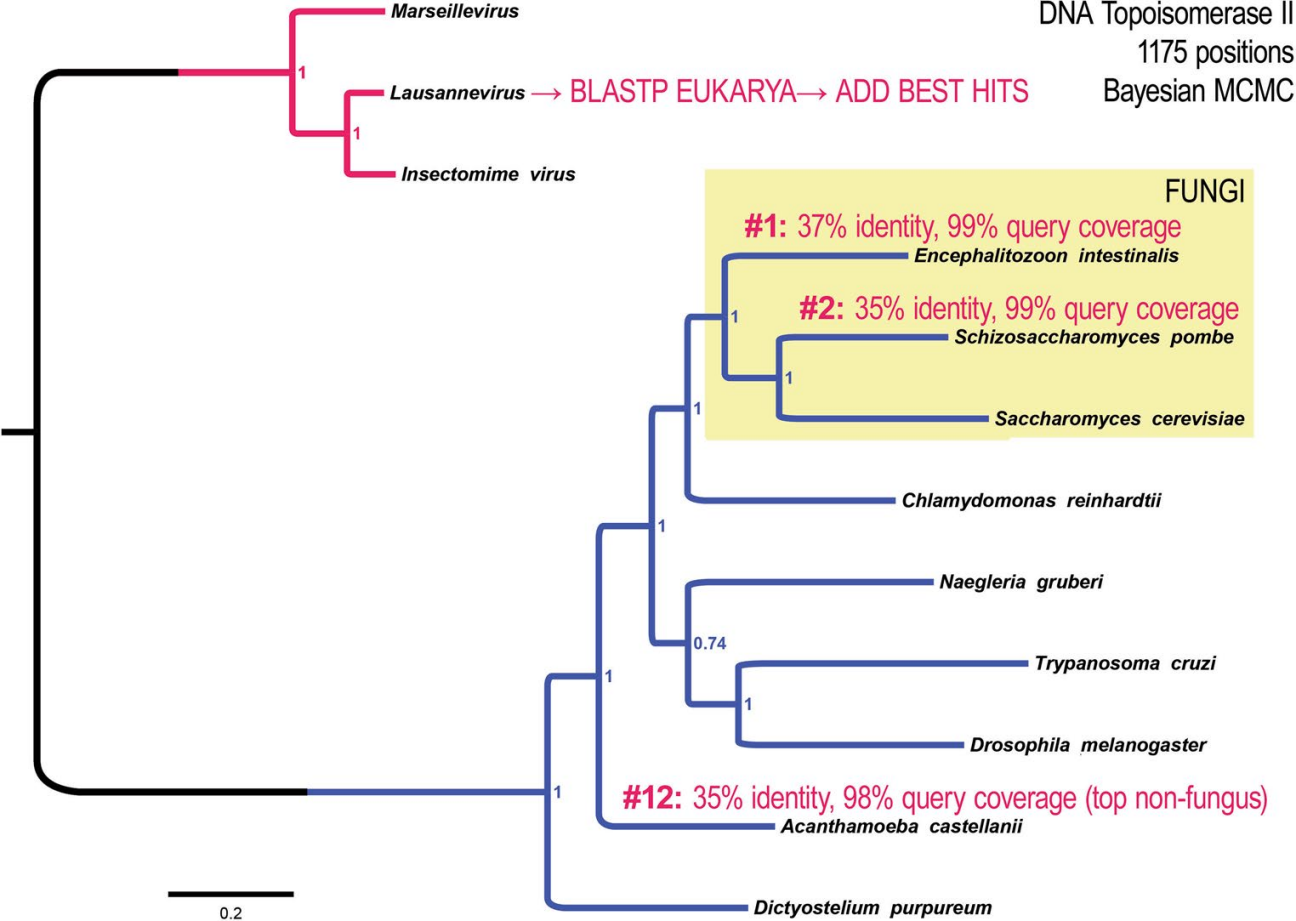
MV DNA topoisomerase II gene is unassignable to any one eukaryotic lineage. The DNA topoisomerase II protein encoded by MV genomes possesses additional domains not seen in the Euryarchaeota DNA gyrase subunit. To determine whether the MV DNA topoisomerase II was derived from a particular eukaryotic lineage, a Bayesian phylogenetic analysis (1 M generations, 25% burn-in of sampling mixed amino acid models, with WAG having complete posterior probability and avg. st. dev. of split freq. = 0.00047) was conducted using top eukaryotic hits from a query using the Lausannevirus DNA topoisomerase II sequence. This identified fungal sequences as the top hits. The top fungal hits (labeled by % identity and % query coverage from the BLASTP query) and the top non-fungal hits were included in the analysis without the archaeal sequences. The results of this analysis are consistent with a model in which the MV DNA topoisomerase II gene was derived from an unknown branch of the stem-eukaryotic (non-eukaryotic) lineage

We conducted a maximum likelihood analysis of all alignment columns with ≥ 90% data (Fig. 3a), as well as Bayesian MCMC analysis (Fig. 3b). Both trees place the MV DNA topo II genes as sister group to Eukarya with Archaea as an out-group (Fig. 3). Thus, these results cannot rule out that the highly conserved DNA topo II gene of Marseilleviridae is derived from a stem-eukaryotic lineage. To further test the idea that the MV topo II gene is not derived from a specific extant eukaryotic lineage, we used the predicted Lausannevirus Topo II protein sequence to search for the most similar eukaryotic homologs. The top eleven hits are from fungi and have nearly perfect query coverage over the nearly 1200 residues with about 37–35% amino acid identity. We then added these top hits to a new alignment with only the eukaryotic and MV taxa and conducted a Bayesian phylogenetic analysis. This expanded analysis, which now includes N-terminal sequences not found in archaeal DNA gyrases, shows that the MV topo II genes were not derived from any one eukaryotic clade (Fig. 4).

## Discussion

**Fig. 5:**
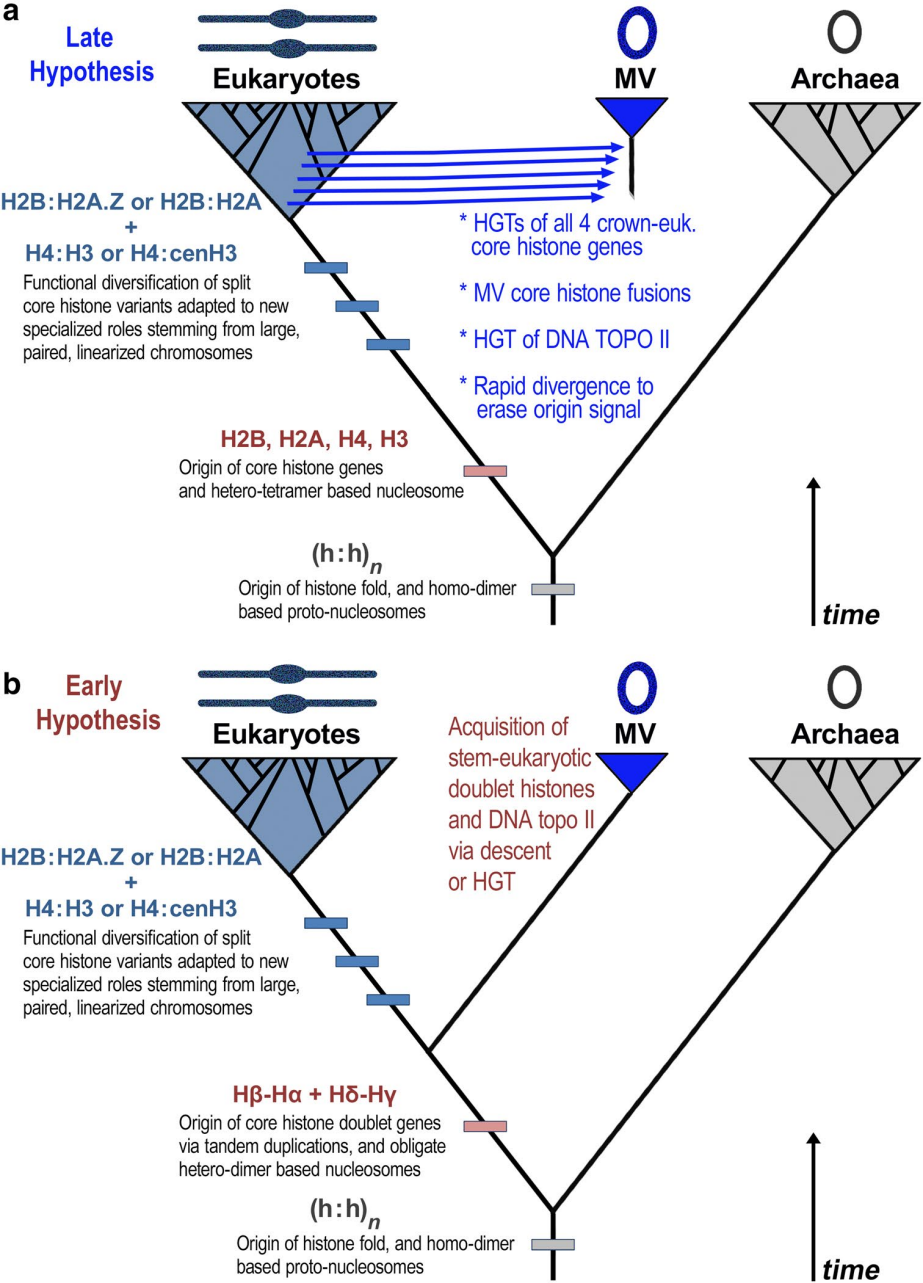
Evolutionary steps toward chromatinized replisomes in eukaryotes and Marseilleviridae. Two hypotheses concerning the evolution of the highly conserved replisome gene repertoires of MV genomes are presented. **a** In the late hypothesis, MV core histone doublets were derived by multiple horizontal gene transfer (HGT) events (blue arrows) from unspecified eukaryotic lineage(s) after eukaryotic diversification. This was followed by fusion of MV core histone genes and evolutionary divergence sufficient to erase sequence affinities with specific eukaryotic lineages as well as specific core histone variant clades. **b** In the early hypothesis, MV core histone doublet genes and DNA topoisomerase genes were acquired from a proto-eukaryotic ancestor encoding fused core histones prior to the evolutionary diversification of eukaryotic core histone variants for H2A and H3. The acquisition from an earlier point in proto-eukaryotic lineage would explain the presence of these genes as fusions because they stem from lineage lacking combinatorial core histone dimer interactions for H2B and H4. It would also explain why they were inherited as a single-core histone locus that evolved by ancient tandem duplications

The presence of a core histone gene repertoire in all MV genomes motivated our desire to test the hypothesis that these genes were acquired by horizontal gene transfer (HGT) from a recognizable eukaryotic lineage and/or a clade of eukaryotic core histone variants (Fig. 5a, “Late” hypothesis). We found no support of any kind for this late HGT hypothesis.

Under the late origin hypothesis, an MV ancestor acquired all four core histone genes by horizontal transfer from a specific eukaryotic lineage or lineages (multiple blue arrows in Fig. 5a). Furthermore, the MV H2A core histone moieties would have come either from a canonical eukaryotic (non-H2A.Z) clade or from a eukaryotic variant H2A.Z clade. Similarly, any MV H3 core histone moiety would have come either from a canonical eukaryotic H3 clade or from a eukaryotic cenH3 clade. Our analyses find no MV affinities for any one these eukaryote-specific gene families, which would have evolved by gene duplication of ancestral core histone genes in late eukaryotic evolution. Thus, this late model would require these genes to diverge sufficiently so as to eradicate any measurable affinities of these MV core histones to their eukaryotic source lineage. This explanation would also have to apply to the larger and highly conserved DNA topoisomerase II protein.

Instead of supporting the late origin hypothesis, we found phylogenetic support for the scenario in which the MV genes encoding the core histone doublets and DNA topoisomerase II enzyme were derived from an earlier part of the stem-eukaryotic lineage that predated neofunctionalization of canonical eukaryotic histone paralogs (see Fig. 5b, “Early” hypothesis). The stem-eukaryotic lineage spans several evolutionary steps (Fig. 5, boxes on stem-eukaryotic branches in A and B) leading to LECA, the Last Eukaryotic Common Ancestor. The MV sequences encoding the core histone moieties and large DNA topo II enzyme are thus likely derived from an early point along the stem-eukaryotic lineage. This point would bisect that lineage into an early branch defined by the evolution of the four core histones (Hα, Hβ, Hγ, and Hδ) that were ancestrally present as doublets, and a later lineage featuring split diversified core histones (e.g., Hβ-Hα → H2B + H2A|H2A.X |H2A.Z|macroH2A, and Hδ-Hγ → H4 + H3|H3.3|cenH3). These results also indicate that the early branch already featured a eukaryote-like DNA topoisomerase II containing additional peptide sequence not seen in archaeal DNA gyrase.

In the later part of the stem-eukaryotic lineage, the evolution of the singlet nature of core histones likely facilitated combinatorial interactions with new specialized variants for genic DNA (H2A.Z and H3), intergenic DNA (H2A and H3), and centromeric DNA (cenH3s). These results can thus be used to infer or at least postulate intermediate steps in the evolution of large, linearized, chromatinized, eukaryotic chromosomes with centromeric pairing (diploidy).

The configuration of a full histone core repertoire in a pair of divergently transcribed histone doublets in MV is remarkable, notwithstanding eukaryotic examples of extreme core histone divergence that are still recognizably eukaryotic. One such example is the repeated loss of centromeric H3s in insect lineages with derived holocentric chromosomes [19]. Another example is the bdelloid rotifer class, which is the largest known clade of animals to be obligately asexual [38, 39]. This class of rotifers substituted high molecular mass H2A variants in place of (i) the canonical H2A histone, which is present in nearly all eukaryotes; and (ii) the H2AX histone, which is involved in eukaryotic DSB repair [40].

## Conclusions

There has been some speculation that new domains of life may be discovered in the era of massive genomic sequencing and analysis [41], but so far this has been mainly served to expand the number of known bacterial phyla [42]. The absence of ribosomal RNAs disallows the ability to use such markers in the case of most viruses, although many NCLDV families possess abundant tRNA repertoires [43]. Nonetheless, there are more eukaryotic protein-fold superfamilies shared only with viruses or only with viruses + bacteria than eukaryotic superfamilies shared only with Archaea or only with Archaea + viruses, respectively [44]. But, some “fourth-domain” viral signals in specific genes of the NCLDV have been proposed [41, 45].

The evidence presented here does not necessarily imply a fourth-domain origin for the shared MV genome, but rather a proto-eukaryotic or stem-eukaryotic origin for the MV repertoire of core histone doublet and DNA topoisomerase II genes. Evidence of a closer relationship between viruses and eukaryotes has already been found in the evolutionary affinity between the HAP2 fusogenic protein underlying eukaryotic gametic fusion, a fundamental aspect of the eukaryotic sexual cycle based on diploid chromosomes, and the class II viral membrane fusogens used by some viruses to fuse to their cellular hosts [46, 47].

Altogether, these findings and other recent results raise additional questions. For example, could the evolution of some large viral genomes have led to core histones functioning in compaction of viral DNA into capsids within its giant replication factories [48], and/or protection of viral DNA from prokaryotic endonucleases? In support of the latter, it is noteworthy that the prokaryotic CRISPR system, which is a dsDNA endonuclease-based anti-viral mechanism in ~ 90% of Archaea and ~ 40% of Bacteria [49, 50], is impeded by the eukaryotic nucleosome [51]. Deciphering exact details of the case or cases will be difficult, given that the vestiges of plausible eukaryotic chromatin origin models are buried by the dynamic evolutionary complexity of eukaryotes, eukaryotic viruses, and in particular the nucleo-cytoplasmic large DNA viruses [2, 16, 52]. Nonetheless, the results presented here may represent a small step in breaking the seemingly long eukaryotic stem branch (see Fig. 5), which culminated in chromatinized linear diploid chromosomes, into intermediate evolutionary stages. This potentiality is worth investigating.

## Methods

### Gene sequences

All sequences were obtained from the following genomes: Cannes 8 virus, complete genome, 374,041-bp circular DNA, Accession: KF261120.1. Melbourne virus isolate 1, complete genome, 369,360-bp circular DNA, Accession: NC_025412.1. Tunis virus fontaine2 strain U484, complete genome, 380,011-bp circular DNA, Accession: KF483846.1. Marseillevirus marseillevirus strain T19, complete genome, 368,454-bp circular DNA,

Accession: NC_013756.1. Lausannevirus, complete genome, 346,754-bp circular DNA, Accession: NC_015326.1. See text for associated publications of each genome. File S1 contains the alignment nexus file for all the core histone moieties analyzed in Fig. 2.

### Phylogenetic analyses

Phylogenetic analysis in Fig. 1a was conducted by the maximum likelihood (ML) method and the Le and Gascuel [8] amino acid substitution model [48]. Phylogenetic analysis in Fig. 1b was conducted using the neighbor-joining method [49] and the JTT matrix-based method [47]. In Fig. 1, the ML and NJ trees were both computed using a ClustalW-based alignment of the concatenated peptide sequences from the H2B-H2A, H4-H3, and H2A-domain-containing (H2ADC) genes found in all MV genomes, and the MEGA6 software package [53]. The MUSCLE (MUltiple Sequence Comparison by Log-Expectation) alignment algorithm and MEGA6 were used to generate protein alignments underlying the analyses of Figs. 2 and 3 [32, 53, 54]. Phylogenetic analysis was conducted using Bayesian MCMC, and mixed amino acid models were tested via MrBayes [29–31]. Sufficient generations were run for the average standard deviation of split runs to be less than 1%. The numbers on the nodes in the Bayesian trees represent posterior probabilities. All other trees give bootstrap replicates and were computed using MEGA6.

## Additional file

**Additional file 1.** Nexus alignment file for the phylogenetic analysis of the core histone protein set.

## Abbreviations

cenH3: centromeric H3 histone
CRISPR: clustered regularly interspaced short palindromic repeats
DNA topo II: DNA topoisomerase II
H2ADC: H2A-domain-containing
HGT: horizontal gene transfer
LECA: last eukaryotic common ancestor
MCMC: Markov chain Monte Carlo
MUSCLE: MUltiple Sequence Comparison by Log-Expectation
MV: Marseilleviridae
NCLDV: nucleo-cytoplasmic large DNA viruses
